# Shelter use interactions of invasive lionfish with commercially and ecologically important native invertebrates on Caribbean coral reefs

**DOI:** 10.1371/journal.pone.0236200

**Published:** 2020-08-26

**Authors:** Christina L. Hunt, Dominic A. Andradi-Brown, Callum J. Hudson, Joshua Bennett-Williams, Frankie Noades, Jocelyn Curtis-Quick, Owen T. Lewis, Dan A. Exton

**Affiliations:** 1 Department of Zoology, University of Oxford, Oxford, United Kingdom; 2 Operation Wallacea, Spilsby, Lincolnshire, United Kingdom; 3 Ocean Conservation, World Wildlife Fund, Washington, D.C, United States of America; 4 School of Environment and Life Sciences, University of Salford, Salford, United Kingdom; 5 Department of Natural Resources and Environmental Sciences, University of Illinois, Urbana, Illinois, United States of America; Institute of Marine Research, NORWAY

## Abstract

Indo-Pacific lionfish have become invasive throughout the western Atlantic. Their predatory effects have been the focus of much research and are suggested to cause declines in native fish abundance and diversity across the invaded range. However, little is known about their non-consumptive effects, or their effects on invertebrates. Lionfish use shelters on the reef, thus there is potential for competition with other shelter-dwelling organisms. We demonstrate similar habitat associations between invasive lionfish, native spiny lobsters (*Panulirus argus*) and native long-spined sea urchins (*Diadema antillarum*), indicating the potential for competition. We then used a laboratory experiment to compare activity and shelter use of each species when alone and when lionfish were paired with each native species. Spiny lobsters increased their activity but did not change their shelter use in the presence of a lionfish, whilst long-spined sea urchins changed neither their activity nor shelter use. However, lionfish reduced their shelter use in the presence of spiny lobsters and long-spined sea urchins. This study highlights the importance not only of testing for the non-consumptive effects of invasive species, but also exploring whether native species exert non-consumptive effects on the invasive.

## Introduction

Invasive predatory species are well known to cause consumptive effects on native species [1–3]. Yet, invasive species may also exert non-consumptive effects, such as competition for food [4] and space [5]. Competition for space occurs in terrestrial [6], freshwater [7] and marine environments [8] and in both plants [9] and sessile and mobile animals [6, 8]. Prior residency may confer an advantage in preventing displacement by a competitor species [9], whereas other factors such as body size may be more important determinants of which species gains access to the shelter [10].

Coral reef ecosystems are in decline around the globe as a result of stressors including climate change [11, 12], unsustainable fishing practises [13], pollution [14] and invasive species [15]. Invasive lionfish have colonised both the western Atlantic [16] and, more recently, the Mediterranean [17]. Lionfish are habitat generalists found on shallow and mesophotic reefs [18, 19], the deep sea [20], mangroves [21], and seagrass beds [22]. Two species of lionfish have invaded the western Atlantic: *Pterois volitans* and *Pterois miles* [23], hereafter collectively referred to as lionfish. The consumptive effects of lionfish on native fish are well known [24], with numerous studies reporting declines in reef fish abundance [25], species richness [26] and recruitment [1]. However, fish are only one component of lionfish diets. Shrimps are also an important prey item [27], particularly in the diet of smaller lionfish [28]. Despite the importance of shrimps in the diet, the consumptive effects of lionfish on shrimps and other crustaceans have been investigated to a lesser extent [29, 30].

In addition to consumptive effects, lionfish are subject to and can exert non-consumptive effects, for example ‘fear effects’, whereby prey change their behaviour to reduce the risk of predation in the presence of a predator [31]. In the western Atlantic, some (but not all) native prey species recognise invasive lionfish as predators [32, 33]. For example, some herbivorous fish show reduced grazing [34, 35] and cleaner shrimp stay closer to their anemones [36] in the presence of lionfish. In addition to exerting non-consumptive effects, lionfish can also be subjected to these effects by humans; lionfish hide deeper in the reef and are more wary of humans as a consequence of lionfish culling programs [37, 38]. Lionfish also exert non-consumptive effects through ‘transmitter’ species, for example lionfish predation on fairy basslet releases blackcap basslet from competition and allows them to occupy prime feeding locations on the reef [39]. Another type of non-consumptive impact is competition. Previous studies have identified competition for prey between invasive lionfish and native grouper [40], potentially affecting native grouper populations. Studies using artificial reefs have demonstrated that limited shelter availability can constrain the abundance of organisms [41], therefore if lionfish occupy shelters on the reef they may exclude native species. This has been suggested by previous studies, indicating that native grouper will avoid a shelter that is occupied by a lionfish [42].

Studies investigating shelter use interactions between lionfish and native invertebrates are lacking. Lionfish show preference for broad-scale complexity such as caves and overhangs [43], which they use as shelter [38], but they avoid fine-scale complexity such as intricate branching corals [43]. Spiny lobsters (*Panulirus argus;* hereafter referred to as lobsters) and long-spined sea urchins (*Diadema antillarum;* hereafter referred to as *Diadema*) are both native invertebrates that use reef shelters. Neither species has been the focus of research on lionfish impacts because spiny lobsters are rarely consumed by lionfish [44] and there are no reports of *Diadema* being consumed by lionfish. Lobster is an economically important species exploited for food across much of its native range [45], whilst *Diadema* is an ecologically important species for its keystone role in macroalgal grazing [46]. Both lobsters and *Diadema* use shelters on the reef to avoid predation [47, 48] and along with lionfish, all three species spend more time using shelter during the day than during the night [49–51]. The availability of reef shelters decreases with increasing shelter size [52], suggesting that shelters at a suitable scale for these three species may be limiting. Research indicating that spiny lobsters and *Diadema* will recruit to artificial shelters that are placed on natural reefs [53, 54] further strengthens our hypothesis that suitable shelters are often limited on coral reefs.

If lionfish show similar shelter preferences as native lobsters and *Diadema*, as seen in grouper-lionfish interactions [55, 56], they may compete for the limited shelters that are available. This could lead to lionfish displacing lobsters and *Diadema* from shelters, thus exposing them to greater predation risk. Similar shelter preferences between lionfish, spiny lobsters and *Diadema* have been suggested [57], and shelter use interactions between lionfish and lobsters are believed to occur. Lionfish are highly abundant by-catch species in lobster traps [58] and the presence of lionfish is associated with lower lobster abundance in both traps [59] and condos (non-enclosed shelter traps; [60, 61]). Competition for shelter between invasive lionfish and native species could therefore have significant impacts on lobster and *Diadema* populations, which may affect economic benefits from the lobster fishery and ecological functions provided by *Diadema*.

In this study we investigate whether invasive lionfish compete with native lobsters and *Diadema* for shelter on Caribbean coral reefs. We first tested whether lionfish, lobster and *Diadema* occupy similar habitats on the reef, by conducting *in situ* assessments of habitat complexity. We then tested whether the presence of lionfish influences the behaviour of lobsters and *Diadema*, using a controlled laboratory experiment with limited shelter availability. Our study clearly identifies the overlap in shelter preferences and illustrates the behavioural responses caused by interactions between native invertebrate species and invasive lionfish, highlighting the importance of non-consumptive impacts of invasive species on the ecology of native species.

## Methods

### Study species and area

Lionfish (assumed to be *Pterois volitans* based on [62]), lobsters (*Panulirus argus*) and *Diadema* (*Diadema antillarum*) were studied in the shallow (3–12 m) near-shore reef system of La Ensenada in Tela Bay, Honduras, at the southern end of the Mesoamerican Reef (S1 Table) from June-August 2019. La Ensenada has a mean coral cover of 8.5% and mean macroalgal cover of 21.4% [54].

### *In situ* habitat associations

Habitat associations of lionfish (*n* = 35), lobsters (*n* = 28) and *Diadema* (*n* = 22) were recorded during roving SCUBA dives at three sites on the La Ensenada reef system (S1 Table). SCUBA dives (*n* = 43) took place between 8:00 am and 2:00 pm across June, July and August. All three species are generally inactive and inside shelter during the day [49, 51, 63], so we assume that the habitat associations observed represent daytime shelters. Habitat was assessed within a 1 x 1 m quadrat that was placed with the centre directly over the individual of interest. Habitat associations were only recorded from individuals that were removed from the reef, thus preventing pseudoreplication, which could have occurred if the same individual was sampled on multiple occasions. We aimed to select animals with body sizes of approximately 16 cm to match the size of the experimental shelter, since many marine species choose shelters that scale with their body size [47, 54]. However, it was not always possible to find animals of this size. Mean sizes (± standard error) of the specimens used for our habitat association study are 21.5 (± 0.5) cm total length for lionfish, 5.5 (± 0.1) cm carapace length for lobsters and 4.8 (± 0.1) cm test diameter for *Diadema*. Details on the conversion from carapace length or test diameter to overall body size are provided in the experimental design section of the methods. These habitat associations were compared to randomly sampled background areas of reef (*n* = 36), obtained by laying three 50 m transects in random directions from the mooring line at approximately 5 m depth at each of the three collection sites (S1 Table). A 1 x 1 m quadrat was placed every 12.5 m along the transects with the measurement as the centre point, omitting the quadrat at 0 m to remove bias in the placement of the start of the transect tape.

**Fig 1:**
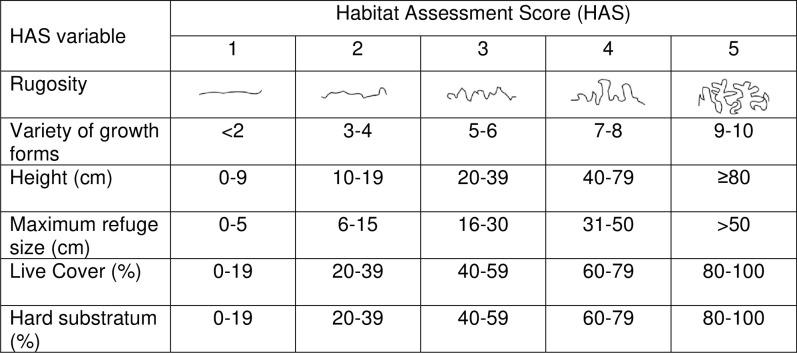
Habitat Assessment Score (HAS) table. This is a modified version of the HAS table [64] that indicates the criteria for each HAS value. Higher HAS values represent greater complexity.

In all quadrats placed, Habitat Assessment Scores (HAS) for six complexity categories were recorded on a scale from 1–5, with larger values indicating higher complexity, following [64]. Maximum refuge size and substratum height were measured, variety of growth forms was counted (this applies to all sessile organisms and includes categories such as encrusting, branching, filamentous, and massive; see [64] for a full list of growth forms) and average live cover (percentage cover of sessile organisms such as live corals, macroalgae and sponges), average hard substratum (percentage cover that was not sand, silt or rubble) and rugosity (the topography of the reef) were estimated ([64]; Fig 1; S2 Table). When aggregations of lionfish or lobsters were encountered, only one quadrat was recorded at the centre of the aggregation to prevent pseudoreplication. No aggregations of *Diadema* were observed.

### Specimen collection

Specimen collections were conducted by recreational SCUBA divers during roving dives in depths of 4.2–12.1 m. The individuals used in our laboratory experiment are a subset of those individuals that were collected during the habitat association aspect of our study. Not all collected individuals were used because the cameras failed during some of the laboratory trials. Lionfish (*n* = 35) were collected from the reef using hand-nets, whilst lobsters (*n* = 28) were caught by hand. Following capture, lionfish and lobsters were transferred to separate 57-litre drybags filled with seawater. *Diadema* (*n* = 22) were collected and transported in a large bucket of seawater. All specimens were slowly brought to the surface and then transported to the laboratory by 15-minute boat ride within the drybag or bucket they were collected in.

### *Laboratory* behaviour experiment

**Fig 2:**
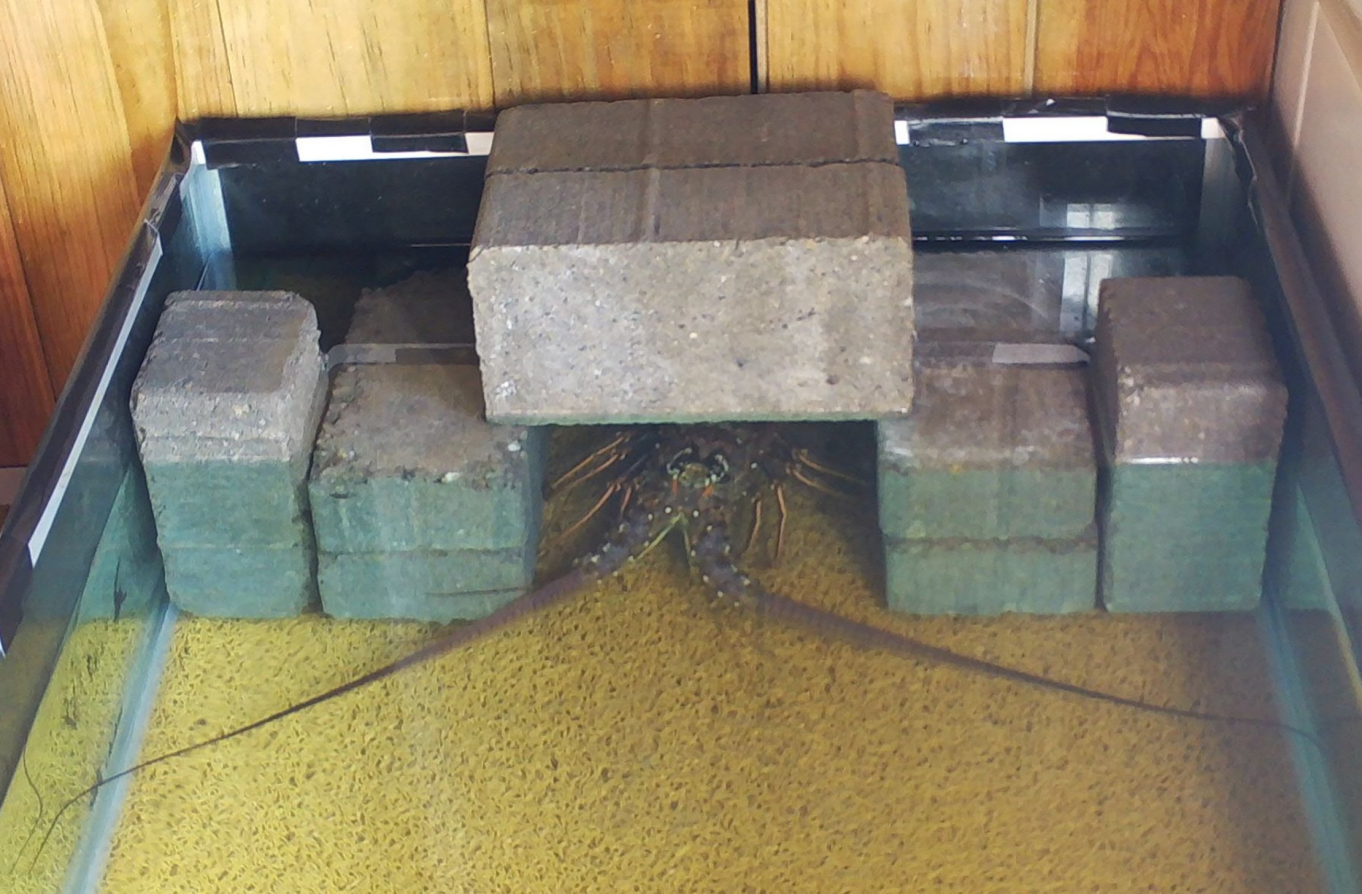
Lobster inside the shelter in the trial tank.

**Aquarium setup.** Three tanks measuring 100 x 55 x 55 cm (length x width x depth) were used for acclimatisation and two tanks measuring 245 × 56 × 24 cm were used to run the trials. We used the largest tanks available to minimise the impact of confinement on interspecific competition [65]. Trial tanks were the same as used those in a lionfish behaviour experiment [43], which are similar or larger than tanks used in published experiments on spiny lobsters [66] and *Diadema* [67]. Tanks were filled with unfiltered natural seawater to a depth of 17 cm. The air conditioning in the laboratory was controlled to ensure that water temperature in the tanks remained at 29°C, matching the ambient sea temperature at the collection sites. Tanks were illuminated from above by sunlight from large windows and thus followed the natural light-dark cycle. Tanks were surrounded by black plastic on four sides to prevent visual disturbance from neighbouring tanks. All acclimatisation and trial tanks contained a shelter that measured 18 x 16 x 15 cm internally (width x height x depth) and was constructed from six concrete bricks (Fig 2). The water level in the tanks extended 0.5 cm above the ceiling of the shelter to ensure that the internal space of the shelter was completely submerged. An internal shelter diameter of approximately 15 cm was chosen to match the previously reported preference of invasive lionfish in Honduras; solitary lionfish most often use 6–15 cm diameter shelters whilst aggregating lionfish most often use 16–30 cm diameter shelters [43]. We aimed to collect animals that matched the internal shelter dimensions to limit shelter sharing and thus create a situation of limited shelter availability.

The shelter was placed at one end of the tank, with the opening facing towards the centre of the tank. Gaps on either side of the shelter were blocked off using extra concrete bricks to prevent animals from sheltering between the edge of the tank and the shelter. Each acclimatisation tank was fitted with a Tetra whisper air pump (Spectrum Brands Inc., Virginia, USA) to maintain oxygenation of the water, however, air pumps were not used in the trial tanks because the much greater surface area would have allowed sufficient oxygen transfer and we did not want the air pumps to interfere with natural diffusion of chemical cues. At the end of every acclimatisation period and every trial, water quality (pH, nitrate, nitrite and ammonium) was tested using a saltwater master test kit and all values were found to be within acceptable water quality guidelines (API Fishcare, Pennsylvania, USA).

#### Specimen acclimatisation

On arrival at the laboratory, each species was placed into a separate acclimatisation tank, with a maximum of two conspecific individuals per tank. Animals were collected between 8:00am and 2:00pm and tested the following morning, resulting in acclimatisation times of 16–24 hours. This was deemed to be enough time because at the beginning of each trial, all individuals were displaying normal day-time behaviour of resting inside shelter [51, 68, 69]. Although shorter acclimatisation times have been used for studies on *Diadema* [67], we chose to standardise the acclimatisation time across species. Animals were not fed during the acclimatisation period to standardise hunger levels between individuals [70, 71].

#### Experimental design

Single individuals of each species were tested alone (lionfish alone *n* = 12, lobster alone *n* = 12, *Diadema* alone *n* = 12) to determine their activity and shelter use in the absence of other, potentially competing, species. Lobsters and *Diadema* were also tested in the presence of a lionfish (lionfish-lobster together *n* = 12, lionfish-*Diadema* together *n* = 7) to determine whether behaviour differed between these ‘together’ trials and the ‘alone’ trials. Each animal was only used once. The individuals used in our laboratory experiment are a sub-set of those used in the habitat association study, thus we now report the range of sizes used for our laboratory experiment. Lionfish ranged in total length (TL) from 14.6–26.6 cm, with a mean (± standard error) of 21.2 (± 0.6) cm. Lionfish size at sexual maturity varies between the sexes and between locations but on average is 10 cm for males and 17.5 cm for females [72], indicating that the majority of our lionfish were likely to be adults. Lobsters ranged in carapace length (CL) from 4.1–7.1 cm, with a mean of 5.4 (± 0.1) cm, and thus were classed as small juveniles [47]. The minimum carapace size at which lobsters can legally be fished is 7.8 cm [61], thus the lobsters in our experiments were below the legal size. Using a CL to TL conversion for *Panulirus argus* [73], we calculated that the lobsters ranged from 10–18 cm in TL. We only used lobsters with hard exoskeletons, indicative of intermoult condition, because crustaceans can be more vulnerable to predation when they have recently moulted [74], potentially influencing their sheltering behaviour in a way that would confuse interpretation of the results. *Diadema* ranged in test diameter from 4.0–5.6 cm, with a mean of 4.9 (± 0.1) cm, and were classified as adults because they had no banded spines [75]. We only used adults of the more common black phenotype because behaviour is known to vary between phenotypes [67]. Using a test diameter to spine length conversion for *Diadema* spp. [76], we calculated that the *Diadema* individuals used in our experiment ranged from 20–28 cm in total diameter.

To begin the trial, lionfish and/or lobsters were placed at the opposite end of the tank to the shelter, but *Diadema*, always began the trial inside the shelter due to a lack of vision. The artificial shelter would not have provided natural olfactory cues for *Diadema*, thus if they had been placed at the opposite end of the tank, they may not have found the shelter. As we were using two identical trial tanks, we randomised which tank was used for each trial.

Trials were filmed using a Bushnell Trophy Cam HD Aggressor (Bushnell Corporation, Missouri, USA) positioned 1 m from the end of the tank containing the shelter and angled down at 45 degrees. This positioning of the camera allowed us to see into the back of the shelter and 17 cm in front of the shelter. The camera filmed using infrared (850 nm wavelength) during both day and night because preliminary trials found that videos were often underexposed when filmed in daylight without the use of flash. Lionfish, lobsters and *Diadema* show low sensitivity to wavelengths above 600 nm [77–79] so the light emitted by these cameras should have been non-detectable. Trials ran for 24 hours and video recordings were made for 60 seconds every hour.

Following the trials, we telson-clipped the lobsters to ensure that individuals were not re-used in subsequent experiments. *Diadema* were not marked because current tagging methods are invasive and can impact survival [80]. Instead, *Diadema* were released by the mooring line at the collection site and subsequent individuals were collected a minimum of 20 m away. Average movement each night by *Diadema* is less than 5 m [81] and individuals often return to the same crevice repeatedly [82], thus we assume that 20 m was sufficient to prevent individuals being recaptured. All lobsters and *Diadema* were released back onto the reef within two hours of the trial ending. Immediately after the trial, lionfish were humanely culled using a two-step method for finfish euthanasia, whereby cervical transection was followed by pithing to destroy the brain tissue [83]. All acclimatisation and trial tanks were emptied, cleaned, and refilled with new seawater between trials.

For each 60 second video, the time spent: (i) active vs resting (activity), and (ii) inside vs outside the shelter (shelter use) were recorded. Individuals were recorded as inside the shelter if at least 25% of their body length was inside and were recorded as active if they were moving across the tank. Lionfish often moved their fins and lobsters moved their legs whilst stationary, but we categorised these behaviours as inactive because the individual remained in one location.

For our analyses, we calculated the time spent in each behaviour over the course of the day, but we removed the first hour of each trial to ensure that all animals had acclimatised to the trial tank for a minimum of one hour. Analysed trial data thus spanned 23 hours–comprised of 23 videos of 60 seconds. We calculated activity as the proportion of time spent active out of the time spent in view of the camera, since when animals were out of view we did not know whether they were active or inactive. We calculated shelter use as the proportion of time spent inside shelter out of the total trial time, since there was only one shelter in the tank so even when not in view of the camera we knew that animals were not inside the shelter (S3 Table). In addition to our formal analyses, we recorded the individual that entered the shelter first and the individual that spent the longest length of time inside the shelter (over the 23-hour trial). In the lionfish-*Diadema* together trials, the *Diadema* always started the trial inside the shelter, as previously explained.

### Statistical analysis

All tests were two-tailed with an *a priori* significance level of 0.05 and were conducted in R version 3.6.2 [84]. All graphs were plotted using the ggplot2 package [85] with the colour-blind friendly viridis colour palette from the viridis package [86].

To visualise *in situ* habitat associations, we performed non-metric multidimensional scaling (NMDS) with a Bray-Curtis dissimilarity matrix using the vegan package in R [87], and plotted vectors of the HAS categories to aid interpretation of the visual output. We then conducted an analysis of similarities (ANOSIM) test to determine whether complexity differed between quadrats (background, lionfish, lobster and *Diadema*).

To examine *in situ* habitat associations for specific HAS categories, we conducted Kruskal-Wallis tests, which were chosen because the HAS data were ordinal, rather than continuous. We conducted one Kruskal-Wallis test for each of the six HAS categories: rugosity, variety of growth forms, substratum height, maximum refuge size, percentage live cover and percentage hard substratum. Each Kruskal-Wallis test compared HAS between background, lionfish, lobster and *Diadema* quadrats. When the results were significant- indicating a difference in HAS value between quadrat types- we ran post-hoc Dunn tests [88] to compare all treatment combinations: background-lionfish, background-lobster, background-*Diadema*, lionfish-lobster, lionfish-*Diadema* and lobster-*Diadema*. Our post-hoc Dunn tests were run using the Benjamini-Hochberg method to correct for multiple testing (by controlling the type I error rate) whilst retaining greater power than the Bonferroni method [89].

Our data for activity and shelter use were proportions. The denominator of the fractions was large (up to 1380), thus allowing the proportions to take a wide range of values. For this reason our data can be described as non-count-based proportions and thus were analysed using beta regression [90], which can account for the heteroskedasticity and skewness that are common with proportion data [91]. Beta regression does not work with values of exactly 0 or 1, so the response variable must be transformed prior to analysis [90, 91]. We transformed our response variables using equation one (modified from [91]) to scale our response between 0.05 and 0.95. We used the default logit link function for the beta regression analyses because the choice of link function will only affect model fit when one or more of the predictors is continuous [90].

**Equation 1.** This equation, modified from [91], converts the response variable y, which is a proportion that can range from 0–1, to a new response variable y’ that can range from 0.05–0.95.

$$y\prime =\frac{(y\times 9)+0.5}{10}$$

Model selection followed the two-step process detailed in [92], whereby we first selected the best model from our candidate models assuming fixed precision. We then created a new series of candidate models that incorporated variable precision, which can account for differences in variance between treatments [90] and thus improve model fit. The best model was deemed to be the one with the lowest AICc value [93]. Most of our models differed from the next best model by more than two AICc units and thus our ‘best’ models have substantial support relative to the next best model [93]. However, two of our models (lobster activity and *Diadema* shelter use) differed from the next best model by less than two AICc units, with the next best model containing one extra parameter. These extra parameters can be considered uninformative [94], thus we selected the simpler models, which had the lowest AICc. We used AICc rather than AIC because of our small sample sizes [93].

Beta regression analyses (hereafter referred to as BR) were conducted using the betareg package in R [91] and AICc values were calculated using the AICcmodavg package in R [95]. Trial type (lionfish alone, lobster alone, *Diadema* alone, lionfish-lobster together, lionfish-*Diadema* together) was always included in the mean models but we also tested whether to include trial tank (left, right) in the mean and precision models. We tested the difference in behaviour (activity and shelter use) between trial types in the 23-hour dataset. Trial tank was never included in the best fitting mean or precision models and thus will not be discussed further.

### Ethics

Lionfish culling was carried out in accordance with the American Veterinary Medical Association Guidelines for the Euthanasia of Animals [83]. All experimental protocols were approved by the University of Oxford Department of Zoology Animal Welfare and Ethical Review Body. No specific permits were required to collect lionfish, lobsters, or urchins. A research permit for this work was obtained from the Instituto de Conservacion Forestal, Honduras (ICF-508-2019).

## Results

### Lionfish, lobsters and *Diadema* have similar habitat preferences

**Fig 3:**
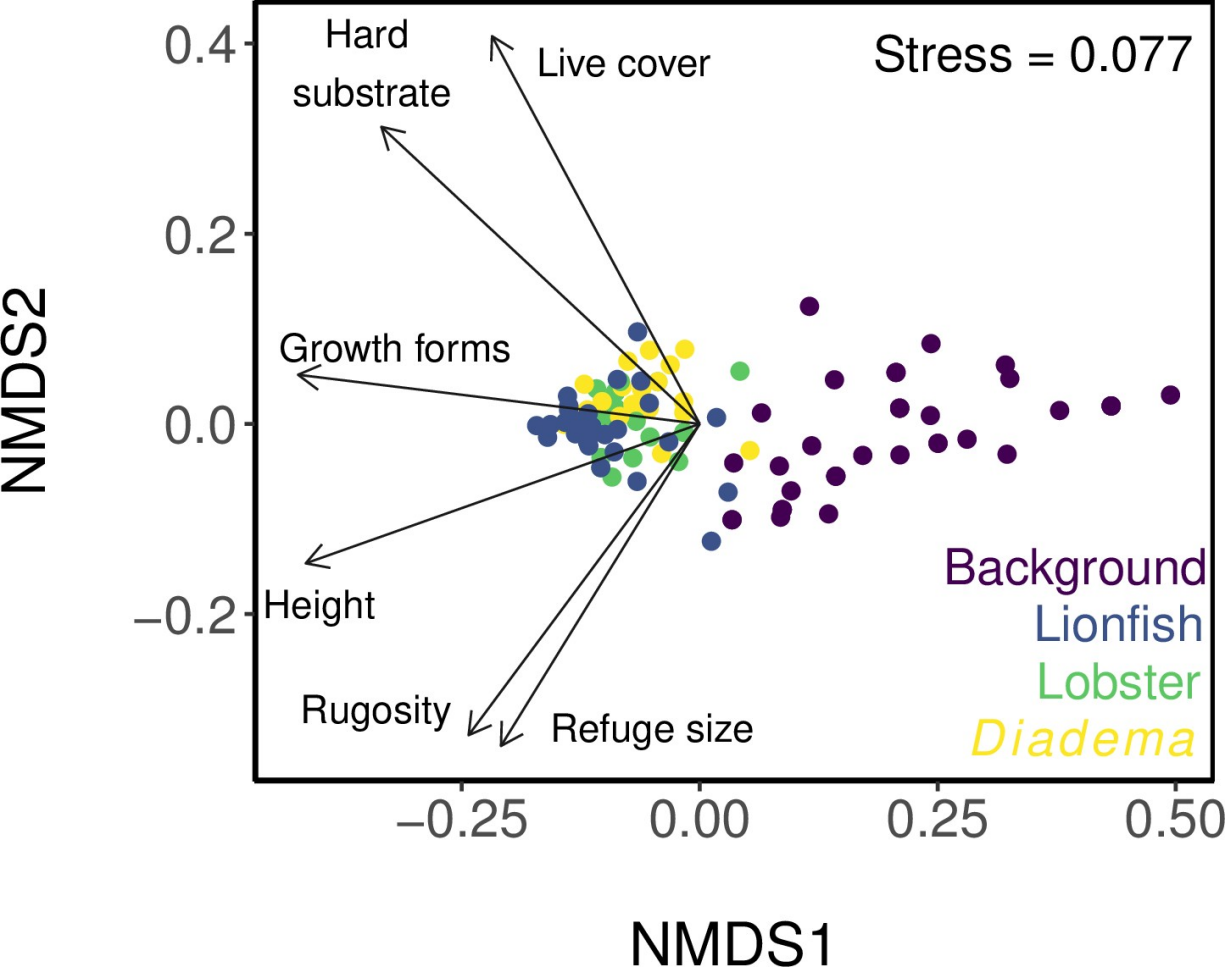
Non-metric multidimensional scaling ordination of reef quadrat complexity. Each point represents a 1 x 1 m quadrat that is either a randomly sampled background quadrat (*n* = 36), or contains a lionfish (*n* = 35), lobster (*n* = 28) or *Diadema* (*n* = 22). The plot was constructed using values of the six HAS categories: rugosity, variety of growth forms, substratum height, maximum refuge size, percentage live cover and percentage hard substratum. Quadrats with more similar complexity are closer together on the plot. Vectors indicate the direction of the gradient for each HAS category.

**Fig 4:**
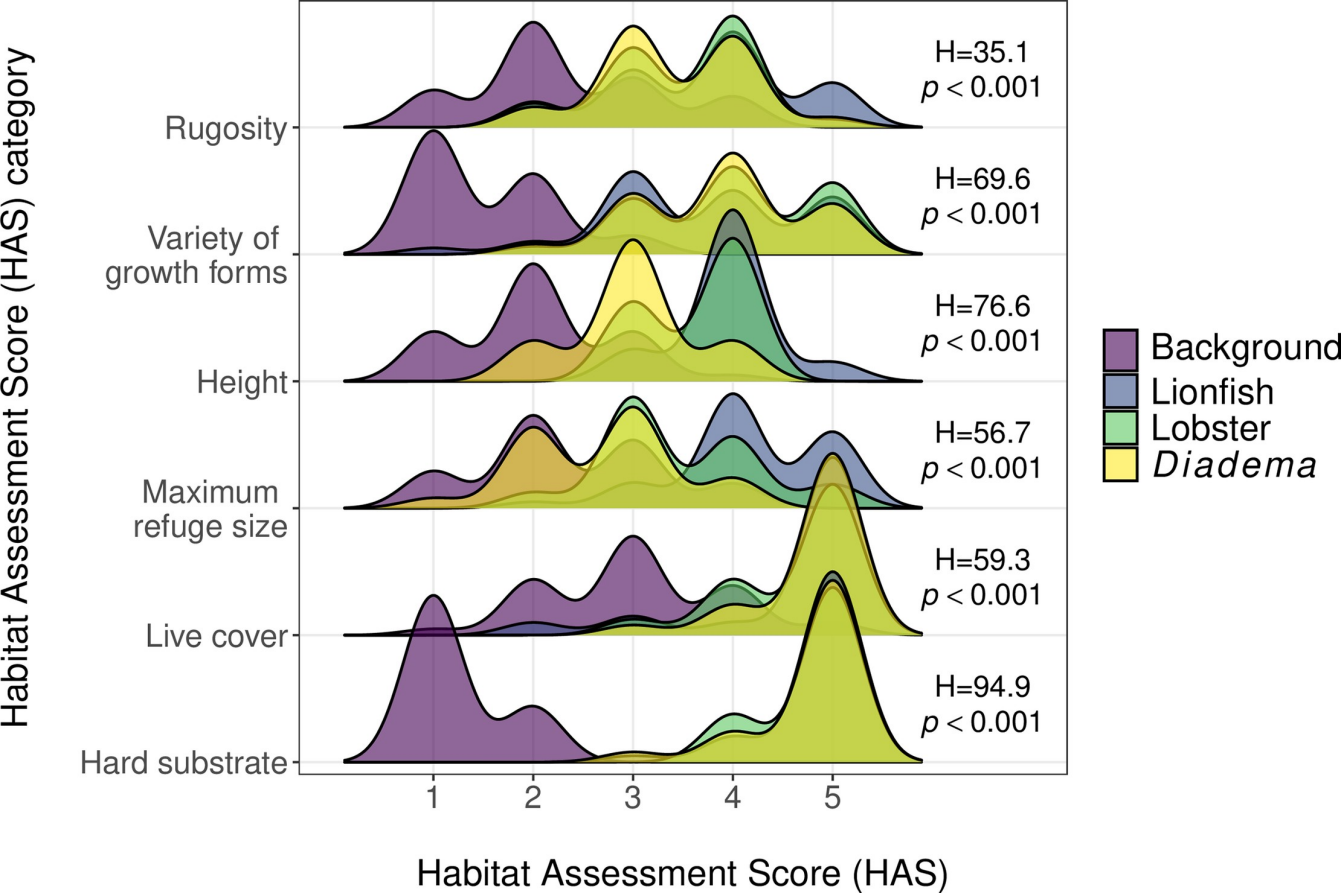
Habitat Assessment Score (HAS) joyplot. HAS are plotted for background (*n* = 36), lionfish (*n* = 35), lobster (*n* = 28) and *Diadema* (*n* = 22) quadrats. Larger HAS represents greater complexity. HAS are recorded on an ordinal scale from 1–5, thus it is the peaks in this joyplot that highlight the overlap, or lack of overlap, between the four groups. H-values and *p*-values represent the test statistic and significance of each Kruskal-Wallis test.

There was a significant difference in habitat complexity between the species quadrats and the background quadrats (ANOSIM, R = 0.440, *p* = 0.001; Fig 3), with all three species generally found in more complex areas of the reef when compared with average reef complexity (Dunn tests, *p*<0.05 for all; Table 1; Fig 4). All three species were found in areas with significantly higher values for rugosity, variety of growth forms, percentage live cover and percentage hard substrate (Dunn tests, *p*<0.05 for all; Table 1), but none of the species differed significantly from one another (Dunn tests, *p*>0.05 for all; Table 1). For height, values for all three species were significantly higher than the background, whilst both lionfish and lobsters were found in significantly more complex habitat than *Diadema* (Dunn tests, *p*<0.05 for all; Table 1).

**Table 1:** Dunn tests results.

		Rugosity		Variety of growth forms		Height		Maximum refuge size		% Live cover		% Hard substrate	
		Z-value	*p*-value	Z-value	*p*-value	Z-value	*p*-value	Z-value	*p*-value	Z-value	*p*-value	Z-value	*p*-value
Comparisons with the background	Background-lionfish	-5.52	<0.001	-6.44	<0.001	-8.17	<0.001	-7.02	<0.001	-6.42	<0.001	-8.34	<0.001
	Background-lobster	-4.28	<0.001	-7.06	<0.001	-6.31	<0.001	-3.94	<0.001	-5.69	<0.001	-7.51	<0.001
	Background- *Diadema*	-3.71	<0.001	-6.17	<0.001	-3.03	0.004	-0.937	0.349	-6.07	<0.001	-7.11	<0.001
Comparisons between species	Lionfish-lobster	0.912	0.434	-0.987	0.486	1.38	0.168	2.66	0.012	0.353	0.724	0.347	1.00
	Lionfish- *Diadema*	1.12	0.393	-0.518	0.725	4.11	<0.001	5.19	<0.001	-0.438	0.794	0.205	1.00
	Lobster- *Diadema*	0.260	0.795	0.383	0.702	2.70	0.008	2.59	0.011	-0.732	0.696	-0.113	0.910

Z-values and Benjamini-Hochberg adjusted *p*-values of post-hoc Dunn tests are displayed for all Habitat Assessment Score (HAS) categories. Highlighted cells represent significant results (*p*<0.05). Z-values are rounded to three significant figures and *p*-values are rounded to three decimal places (except for *p*<0.001).

Maximum refuge size showed the greatest difference between species. Lionfish were found in areas with greater maximum refuge sizes than lobsters or *Diadema*, and greater than the background (Dunn tests, *p*<0.05 for all; Table 1). Lobsters were found in significantly more complex habitat than *Diadema* or the background (Dunn tests, *p*<0.05 for both; Table 1). *Diadema* did not differ significantly from the background (Dunn test, Z = -0.937, *p* = 0.349). The median refuge size HAS were: background (6–15 cm), *Diadema* (16–30 cm), lobsters (16–30 cm) and lionfish (31–50 cm). Despite these differences between species, there was still considerable overlap in HAS for maximum refuge size between species (Fig 4).

### Lionfish exert non-consumptive effects, but so do native species

**Fig 5:**
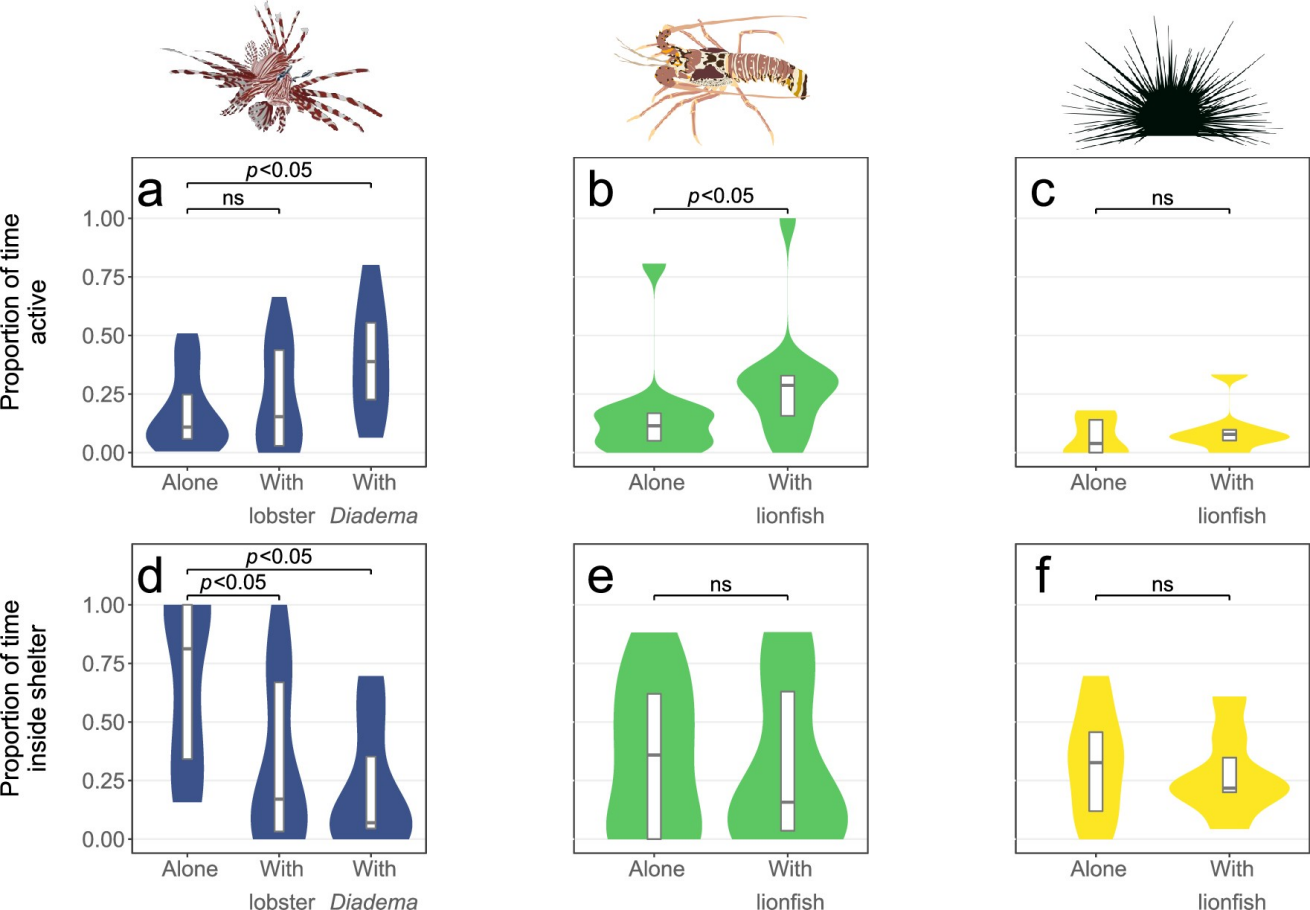
Activity and shelter use of lionfish, lobsters and *Diadema*. The proportion of time that lionfish, lobsters and *Diadema* spent active (a,b,c) and inside shelter (d,e,f) in the ‘alone’ and ‘together’ treatments during the 23-hour trial. Each plot represents how the behaviour of the focal species (indicated by the creature image) varies between the ‘alone’ and ‘together’ trials. For the lionfish graphs (a and d), the ‘alone’ bar represents the behaviour of lionfish when alone, the ‘with lobster’ bar represents the behaviour of lionfish in the presence of a lobster, and the ‘with *Diadema*’ bar represents the behaviour of lionfish in the presence of a *Diadema*. For the lobster (b and e) and *Diadema* (c and f) graphs, the ‘alone’ bar represents their behaviour when alone, whilst the ‘with lionfish’ bar represents their behaviour in the presence of a lionfish. Violin plots represent the spread of the data and are scaled so that the violins on each plot have the same area. Each violin plot is overlaid with a boxplot showing the median and inter-quartile range. All treatments have *n* = 12, except the ‘lionfish-*Diadema* together’ treatment, for which *n* = 7.

Lobsters were 1.8 times more active (BR, Z = 2.43, *p* = 0.015; Fig 5), but did not change their shelter use (BR, Z = -0.119, *p* = 0.906), in the presence of a lionfish during the 23-hour trial. The presence of lionfish had no effect on activity (BR, Z = 1.77, *p* = 0.077; Fig 5) or shelter use (BR, Z = 0.302, *p* = 0.763) of *Diadema*. Lionfish and lobsters shared the shelter in one out of twelve trials (8% trials), whilst lionfish and *Diadema* shared the shelter in two out of seven trials (29% of trials). In ten of the twelve lionfish-lobster together trials (83% trials), the species that entered the shelter first was also the species that spent the most time inside the shelter (Table 2). However, in the lionfish-*Diadema* together trials, lionfish and *Diadema* appeared equally likely to occupy the shelter (dominating the shelter in *n* = 3 and *n* = 4 trials respectively), even though *Diadema* always began the trial inside the shelter.

**Table 2:** Shelter occupancy data from lionfish-lobster together trials.

Species that entered the shelter first	Species that spent the most time inside the shelter	
	Lionfish	Lobster
Lionfish	6	1
Lobster	1	4

Trials (*n* = 12) are categorised based on which species entered the shelter first and which species spent the most time inside the shelter.

Lionfish were 1.9 times more active in the presence of *Diadema* than when alone (BR, Z = 2.39, *p* = 0.017; Fig 5) during the 23-hour trial. In contrast, the presence of lobsters had no effect on lionfish activity (BR, Z = 0.487, *p* = 0.626).Lionfish used shelter 1.9 times more when alone than when in the presence of lobsters (BR, Z = -2.75, *p* = 0.006)and 2.2 times more when alone than when in the presence of *Diadema* (BR, Z = -2.85, *p* = 0.004).

## Discussion

We found invasive lionfish have similar *in situ* habitat preferences as native lobsters and *Diadema* (Fig 4), thus creating the potential for competition when shelter availability is limited. Our experimental trials then indicated that lionfish presence led to increased activity in lobsters but had no effect on *Diadema* (Fig 5). We found the presence of lionfish did not affect shelter use by lobsters or *Diadema*. Lionfish, however, had reduced shelter use when in the presence of lobsters or *Diadema*.

### Lionfish, lobsters and *Diadema* preferred high complexity habitats

All three species preferred habitat that was more complex than the background average. For lobsters and *Diadema*, higher complexity habitat provides protection from predation [47, 48]. The preference of lionfish for complex habitat applies across the invaded western Atlantic range (e.g [57, 96, 97]). In areas with frequent culling, lionfish use complex habitat to escape culling by humans [38]. However, lionfish have few natural predators in the invaded range [98], so in areas where they are not culled by divers, habitat preference may instead be related to hunting efficiency [43]. For four of the HAS metrics (rugosity, variety of growth forms, percentage live cover and percentage hard substrate) all three species showed similar habitat preference. This suggests lionfish, lobsters and *Diadema* are likely to compete for reef shelters in the Caribbean. Reef flattening, a reduction in hard coral cover and subsequent decline in architectural complexity, is occurring across the region [99], reducing the availability of shelters and so leading to increased shelter competition.

We found significant differences in maximum refuge size between species. A previous study from Honduras found that lionfish preferred shelter sizes of 6–15 cm and 16–30 cm [43]. However, we found that lionfish preferred shelter sizes of 31–50 cm. This disparity may reflect differences between the availability of shelters on different reefs, with lionfish habitat associations reflecting their preferred habitat based local habitat availability. Spiny lobsters use shelters that scale to their body size when alone [47] but they will use larger shelters when aggregating with conspecifics [100]. Most of the lobsters we found were solitary, which may explain their preference for smaller shelters of the size 16–30 cm. When suitably sized shelters are unavailable, spiny lobsters will use any crevice that is large enough to protect their abdomen [50]. Although we attempted to collect similar sized individuals, the lionfish we collected were larger on average than the lobsters, which may explain the observed difference in preferred shelter size. We used lobsters that were below the minimum legal size, which suggests that the shelter size preference of larger, legal-sized, lobsters may overlap more with that of lionfish. A study on *Diadema* in Utila, Honduras, found that they preferred refuges of the size 5–15 cm [54], which is smaller than the median refuge size of 16–30 cm found in our study. *Diadema* select shelters that scale with their test size to avoid predation [54], therefore this difference in shelter size preference may be related to the high proportion of juvenile urchins present at the sites surveyed by Bodmer (54),whereas we only included adults in our study. By converting lobster carapace size and *Diadema* test diameter to total body size, we found that the *Diadema* were larger in overall size than the lobsters and lionfish. Although *Diadema* were larger, it is likely that they chose smaller shelters than lionfish or lobsters because they choose shelters that scale with their test diameter [54], rather than with their total body size. This indicates that *Diadema* may only be subject to shelter competition from small lionfish.

### The relationship between lionfish and native invertebrates

Lobsters became more active in the presence of lionfish. This increase in activity may be related to aggression, as spiny lobsters display several aggressive behaviours [101]. American lobsters (*Homarus americanus*) spend more time active in the presence of other lobsters than when alone and this activity is related to fighting and defending a shelter [102], so the increased activity in our study may be related to shelter defence. Increased activity can reduce the scope for growth in other crustaceans [103], and so could result in reduced growth rates of lobsters. In the absence of shelter, spiny lobsters will remain motionless in the presence of a predator [104], suggesting that increased activity may attract predators and increase predation risk. Lobsters sense their environment using olfactory [66], visual [105] and tactile [106] cues and can detect predator olfactory cues from at least 2 m away [107]. Our experiment cannot distinguish which cues might have been used by the lobsters to detect lionfish, but if they used olfactory cues then the results we observed may be widespread on reefs with invasive lionfish populations, even when lionfish are out of visual range of the lobster. We did not conduct trials with two lobsters, or trials where a lobster was paired with another species, thus we cannot conclusively show that the increased activity of lobsters is driven by the presence of lionfish. Even if the observed response is not specific to lionfish presence, the increase in activity is still cause for concern because as lionfish populations increase, the frequency and duration of lionfish-lobster encounters is likely to increase.

Despite the increase in activity, there was no change in shelter use in the presence of a lionfish, suggesting that the lobsters may be dominant and able to displace lionfish from the shelter, or prevent displacement by lionfish once they are inside the shelter. The lack of change in shelter use suggests that the increased activity observed in the presence of a lionfish is unlikely to be related to submissive behaviour and movement away from the shelter. Lionfish and lobsters shared the shelter in one of our trials, suggesting that even on reefs with limited, and already occupied, shelters, lobsters may still be able to access the shelters. However, we found that prior occupancy influenced shelter use, as in other fish and crustaceans [108, 109]. We found that the first species to enter the shelter was generally the species that spent the most time in the shelter during the trial. This suggests that if shelters are not large enough for lionfish and lobsters to share, then prior occupancy of a lionfish may prevent shelter use by lobsters.

Lionfish had no effect on activity or shelter use of *Diadema*. Several fish families and known predators of *Diadema* [75] and predator cues trigger increased movement [110], so the lack of activity change suggests that lionfish were not considered to be a predator. Alternatively, the lack of a change in activity or shelter use may be because the experimental shelter was considered high quality, as *Diadema* are less likely to flee from a high-quality crevice [82]. In the absence of predators, *Diadema* will avoid shelters containing conspecific odours, which is thought to be a method of avoiding shelter competition [110]. Lionfish and *Diadema* shared the shelter in two of our seven trials, suggesting that lionfish are not considered a shelter competitor and thus the presence of a lionfish inside the shelter does not impact *Diadema* shelter use. However, we found that prior occupancy had no effect on which species used the shelter the most, suggesting that in some cases lionfish may be able to exclude *Diadema* from their shelters.

### Impacts of lobsters and *Diadema* on lionfish

Lionfish did not change their activity in the presence of lobsters but they became more active in the presence of *Diadema*. Lionfish increase their activity in response to intraspecific competition [49], thus we suggest that lionfish may become more active in the presence of *Diadema* because they perceive them as a shelter competitor. Lionfish may not have increased their activity in response to lobsters because the lobsters may have been perceived as a dominant competitor, as is suggested by other studies on shelter use interactions between fish and crustaceans [111, 112]. The differential response of lionfish to lobsters and *Diadema* indicates that lionfish are not simply responding to over-crowding (there are two individuals in the ‘together’ trials but only a single individual in the ‘alone trials). Instead, lionfish behavioural responses are determined by the identity of the competitor, as is observed in other fish species [113].

Increased activity in lionfish may lead to less energy being available for other functions such as growth. In areas where lionfish are culled by divers, increased activity of lionfish may make them more conspicuous and easier to capture, thus aiding culling efforts. But an increase in lionfish activity may not always be beneficial for the native community. Increased activity may increase the likelihood of interactions with native species, potentially leading to other non-consumptive effects, such as reduced grazing by herbivorous fish [34, 35].

In contrast to other invasive species, where shelter use of the invasive is unchanged in the presence of native species [42, 111], we found that lionfish reduced their shelter use in the presence of lobsters or *Diadema*. Our findings lend support to anecdotal evidence that reefs with high urchin densities support few lionfish [57], potentially because urchins occupy all available shelters. In our lionfish-lobster together trials we found an effect of prior residency, where lionfish were less likely to dominate a shelter if a lobster occupied the shelter first. However, the shelter we used in our laboratory experiment (16 cm) was smaller than the preferred shelter size of the lionfish used in this study (31–50 cm), thus the motivation of the lionfish to use the shelter may have been less that it would be had the shelter been larger.Nevertheless, reduction in shelter use by lionfish may mean that if shelter is limiting, it is the natives that gain access to the shelters. Reduced shelter use may be detrimental to lionfish health and survival, meaning that lionfish may be less successful invaders on reefs with limited shelter. Feeding success may be reduced if use of shelters enhances feeding success, as suggested by [43], thus leading to reduced consumptive effects of lionfish on reef communities. Larger and heavier female lionfish produce more eggs [114], so any subsequent loss in body condition may reduce fecundity and thus reduce the population growth rate. Reduced shelter use of lionfish may therefore be beneficial to reef communities.

### Management implications

Studies on the commercial spiny lobster fishery have shown that lionfish are often caught as by-catch in traps [58] and the presence of lionfish is associated with reduced lobster abundance in traps [59] and condos (non-enclosed shelter traps; [60, 61]). These results have led to the suggestion that lionfish may deter or out-compete lobsters [59]. Competition between lionfish and lobster may be particularly strong around lobster condos because these are often placed in areas of otherwise low habitat complexity [60]. In our experiments we found that lionfish, rather than lobster, reduced their shelter use when the two species were together, suggesting that lobsters may instead out-compete lionfish. We hypothesise that lobsters exclude lionfish from traps and condos and that the strength of the behavioural change in lionfish may be related to lobster abundance in the trap. This would explain why lionfish are found in traps with small, but not large, numbers of lobsters. The lobsters used in our experiments were below the legal harvesting limit of 7.8 cm carapace length [61] and so our results may not be applicable to larger lobsters. However, if larger lobsters are stronger shelter competitors, as in other lobster species [115], then the effects on lionfish shelter use that we observed may be even stronger when lionfish compete with larger lobsters.

Although our results suggest that lionfish do not affect lobster shelter use, and therefore may not reduce lobster catches, this does not mean that lionfish have no effect on spiny lobster fisheries. The presence of lionfish in lobster condos increases handling time by fishers as they work more cautiously around lionfish to avoid envenomation and this may lead to reduced earnings as fishers are unable to visit as many condos each day [61]. However, these reduced earnings could be partially compensated by increasing lionfish fisheries and selling the lionfish to restaurants or developing value-added lionfish products–such as jewellery [61, 116].

A lack of structural complexity and the subsequent lack of predation refugia has been suggested as a barrier to recovery of *Diadema* following the 1983–1984 mass mortality [117]. One restoration effort has thus been to provide artificial shelters [54]. Lionfish often use and aggregate around artificial structures [118, 119] and could potentially hinder restoration efforts if they affect *Diadema* shelter use. We initially hypothesised that lionfish would enter the shelter and exclude the *Diadema*, but our results showed that lionfish had no effect on *Diadema* activity or shelter use. This lack of response by *Diadema* suggests that lionfish may not interfere with restoration efforts and that the presence of lionfish on a reef may not a barrier to recovery of *Diadema*.

Lobster traps may prove useful for lionfish management, as studies have shown catches of almost 3000 lionfish in a single lobster fishing season [58]. Trapping will complement current management methods because culling by recreational divers is restricted to 30 m [37], but traps can be deployed much deeper to target the larger and more mature lionfish that are not currently culled [37]. Our results have shown reduced shelter use of lionfish in the presence of lobsters, suggesting that lionfish catches would be maximised in the absence of lobsters. To develop lionfish-specific traps, current lobster traps need to be modified to increase lionfish catch but reduce lobster catch. Some work has been done in this area [120], but lionfish-specific traps are still being tested [121] and are not yet commercially available. Current diver-based culling is insufficient to satisfy demand for international export of lionfish [116], but the development of a lionfish trap fishery could provide a low cost removal method that can be applied to depths inaccessible to divers [18].

## Conclusion

Our results have demonstrated the importance of testing for non-consumptive effects of invasive species. We found that lionfish are not detrimental to all native species, as there was no effect of lionfish on *Diadema* activity or shelter use. Invasive species research often focuses on the impacts on the invaded community or on trophic interactions exerted by native species on invasive species, however, we have shown the importance of investigating non-consumptive effects of the native species on the invasive. These effects may prove detrimental to the invader or may lead to additional negative effects on native species. By studying the interactions between lionfish and two different invertebrates, we have demonstrated that non-consumptive effects of native species on invasive species may be widespread. Our research suggests that we should look beyond the consumptive effects of invasive predators and consider how native species may induce changes in invasive species behaviour.

## Supporting information

S1 Table Dive site co-ordinates. Co-ordinates of the mooring lines at each of our three study sites in Tela Bay, Honduras. Lionfish (*n* = 35), lobsters (*n* = 28) and *Diadema* (*n* = 22) were collected from all three sites for use in the laboratory experiments. We recorded Habitat Assessment Scores (HAS) for 12 background quadrats at each site and for each individual that was collected. A total of 43 SCUBA dives took place between 8:00 am and 2:00 pm across June, July and August 2019. Coordinates are given in WGS84.
(CSV)

S2 Table Habitat Assessment Score (HAS) data. Six HAS values were recorded for each quadrat containing lionfish (*n* = 35), lobsters (*n* = 28) and *Diadema* (*n* = 22) and for randomly sampled background quadrats (*n* = 36). HAS values range from 1 (low complexity) to 5 (high complexity). Date, site and animal size are also provided.
(CSV)

S3 Table Raw data and summary values of activity and shelter use across each 23-hour trial. 23 observations of 60 seconds were recorded during each 23-hour trial, thus each trial comprises 1380 seconds of observation. The time that each animal spent active in view of the camera, inactive in view of the camera and inside shelter is shown in seconds (columns G-O). Time spent active was divided by total time in view of the camera (time active + time inactive) to give proportion of time active (columns Q, S and U). Time spent active or inactive was only recorded when the individual was in view of the camera, thus time active + time inactive is not necessarily equal to total trial time (1380 seconds).Time spent inside shelter was divided by total trial time (1380 seconds) to give proportion of time inside shelter (columns W,Y and AA). These proportions were than transformed using equation one to remove any values of 0 or 1 (these transformed proportions are labelled proportion_no01; columns R, T, V, X, Z and AB). Total length of lionfish, lobster carapace length and *Diadema* test diameter are also provided (columns D, E and F).
(CSV)
